# Effects of an early transfer from incubator to a warming crib in very low birthweight preterm infants

**DOI:** 10.1186/s12887-024-04795-y

**Published:** 2024-05-09

**Authors:** Sandra Greve, Nora Bruns, Anne-Kathrin Dathe, Michael M. Schuendeln, Ursula Felderhoff-Mueser, Anja Stein

**Affiliations:** 1 Department of Paediatrics I, Neonatology, Paediatric Intensive Care and Paediatric Neurology, Universitätsmedizin Essen, University of Duisburg-Essen, North-Rhine Westphalia, 45122 Essen, Germany; 2 Department of Paediatrics III, Department of Pediatric Hematology and Oncology, Universitätsmedizin Essen, University of Duisburg-Essen, North-Rhine Westphalia, 45122 Essen, Germany; 3 Department of Health and Nursing, Occupational Therapy, Ernst-Abbe-University of Applied Sciences, Jena, Germany

**Keywords:** Preterm infant, Thermoregulation, Incubator, Warming crib, Length of stay

## Abstract

**Purpose:**

Very low birth weight infants are cared for postnatally in the incubator because of adverse consequences of hypothermia. Data on the optimal weight of transfer to a warming crib are rare. The aim of this study was to determine the course of temperature and body weight during a standardized transfer to a warming crib at a set weight.

**Methods:**

Prospective intervention study in very low birthweight infants who were transferred from the incubator to a warming crib at a current weight between 1500 g and 1650 g.

**Results:**

No infant had to be transferred back to an incubator. Length of hospital stay was equal compared to a historical cohort from the two years directly before the intervention. The intervention group showed an increase in the volume fed orally on the day after transfer to the warming crib, although this did not translate into an earlier discontinuation of gavage feedings. Compared to the historical group, infants in the intervention group could be transferred to an unheated crib at an earlier postmenstrual age and weight.

**Conclusions:**

Early transfer from the incubator to a warming crib between 1500 g and 1650 g is feasible and not associated with adverse short-term events or outcomes.

**Trial registration:**

DRKS-IDDRKS00031832.

## Background

8% of infants in Germany in 2020 were born prematurely. According to data from the German Institut für Qualitätssicherung [1], 0.66% of all infants were born at a very low birthweight below 1500 g (VLBW). The neonatal units at the University Hospital in Essen admit 650 to 700 neonates annually. During the study period, our neonatal intensive care unit treated 117 VLBW infants. On average, VLBW infants constitute approximately 12% of all neonatal patients in our hospital, underscoring the significance of this patient cohort.

A lack of subcutaneous adipose tissue causes increased energy expenditure to maintain the body temperature, especially in VLBW infants. Hypothermia can lead to hypoglycemia, respiratory distress, and ultimately tissue hypoxia. Therefore, the primary goal for these infants is to provide thermoneutral care. In an incubator, heat loss due to conduction, convection, radiation, and evaporation are reduced to a minimum [2]. During the hospital stay, infants are gradually weaned from incubator conditions to an open crib with warming mattress (warming crib). From there, the heat is gradually reduced until the infant tolerates an open unheated crib, which is one of the conditions necessary for discharge.

Clinical practice regarding weight limits at which preterm infants are transferred from the incubator to the warming bed varies widely between different countries and centers [3–9]. Several studies [4, 5, 7, 9, 10] showed that preterm infants can maintain a stable body temperature when removed from the incubator at a body weight as low as 1600 g. In three studies, earlier transfer to the warming crib was associated with a shorter hospital stay [6, 9, 10].

The aim of this monocentric prospective study was to determine the course of temperature and body weight during a standardized transfer of 42 VLBW infants from the incubator to a warming crib at a current weight between 1500 g and 1650 g. Infants were compared to a historical control group that did not differ in birth weight and gestational age but were transferred to the warming crib at a higher body weight (1710 ± 126 g). Furthermore, feeding behavior, weight development, and length of hospital stay were compared between the two groups.

## Methods

This is a prospective intervention study involving very low birthweight infants who were transferred from the incubator to a warming crib when their current weight ranged between 1500 g and 1650 g. We observed a notable shift in management practices, characterized by a trend towards lower weight thresholds for transitioning from the incubator to the warming crib. To assess the safety of this approach, we conducted a study with standardized weight criterion for transfer.

### Eligibility

Infants born at the University Hospital Essen between December 2012 and September 2014, with a birthweight < 1500 g, regardless of postmenstrual age (PMA), were eligible for the study when they reached a current weight between 1500 g and 1650 g. Patient characteristics are shown at Table 1.

**Table 1:** Patient characteristics of intervention (IV) and historical control (HC) group

	IV *n* = 42	HC *n* = 42	*p*-value
male, n (%)	23 (54.7%)	21 (50.0%)	
birth weight [g]	1008 ± 275	1102 ± 294	0.252
	[530–1450]	[460–1540]	
PMA at birth	28.2 ± 2.3	28.6 ± 2.1	0.498
	]23.9–32.3]	]23.3–32.1]	
weight [g] at transfer	1558 ± 46	1710 ± 126	< 0.001
	[1490–1650]	[1540–1990]	
PMA at transfer	33.1 ± 1	33.9 ± 2	0.005
	[30.7–36.1]	[31.6–39.4]	

Notes. Data are presented as mean ± standard deviation [range], if not indicated otherwise

g = gramm, PMA = postmenstrual age

The infant had to be clinically stable, not requiring observation in the incubator. Exclusion criteria included invasive mechanical ventilation or noninvasive respiratory support, such as binasal continuous positive airway pressure (CPAP), with an additional oxygen requirement of > 0.25, failure to regain birth weight at the time of screening, ongoing phototherapy, or an indwelling central venous catheter / umbilical catheter.

### Termination criteria

Inability to maintain body temperature above 36.5 °C, was an indication to transfer back to the incubator, as was clinical instability (e.g., increased episodes of apnea and bradycardia), clinical or laboratory signs of infection, or the need for phototherapy according to the standard of care at our center.

### Patient recruitment

Parents of VLBW infants were approached when the infant reached a weight of 1400 g, and written informed consent was obtained (Table 1).

### Outcomes

The primary outcome was the successful transfer to a warming bed without hypothermia during the first 72 h following transfer in the intervention group (IV group).

Secondary outcomes included the length of hospital stay, postmenstrual age (PMA) at transfer to a normal bed (± 2 days), at discharge (± 2 days), and when reaching full oral feedings, as well as body weight at several time points (before and after transfer to the warming bed, at a PMA of 36.0 weeks (± 2 days), at transfer to a normal bed, at the time of discharge (± 2 days), and at term equivalent age (± 2 days) (refer to Table 2). If infants were discharged before term equivalent age, data were collected at readmission, which was routinely scheduled for vaccination and MRI at term equivalent age. Additional secondary outcomes in the IV group included stability of vital signs within the first 72 h after transfer and the amount of oral feeding.

**Table 2:** results of intervention (IV) and historical control (HC) group

		IV *n* = 42 mean (95% CI)	HC *n* = 42 mean (95% CI)
Weight gain	48 h before (g/kg/d)	24 ± 17^2^ (18–29)	18 ± 11^11^ (14–22)
	48 h after (g/kg/d)	20 ± 16^9^ (15–26)	16 ± 14^10^ (11–21)
end caffeine medication	DOL	51 ± 28^1^ (42–59)	48 ± 28^2^ (38–57)
	PMA weeks	35.6 ± 3^1^	35.0 ± 3^2^
unheated crib	PMA weeks	35.5 ± 1^3^ (35.0–35.9)	36.3 ± 2^4^ (35.6–36.9)
	weight (g)	2057 ± 211^3^ (1988–2124)	2233 ± 251^1^ (2153–2313)
weight	PMA 36.0 (g)	2130 ± 293^5^ (2025–2236)	2189 ± 251^6^ (2105–2272)
	PMA 40.0 (g)	3250 ± 518^6^ (3076–3421)	3133 ± 452^7^ (2980–3286)
	discharge (g)	2768 ± 604^1^ (2575–2961)	2747 ± 740^1^ (2511–2983)
length of stay	days	73 ± 33^8^ (63–84)	71 ± 36 (60–82)
end of gavage feeding	PMA in weeks	35.5 ± 2^6^ (34.7–36.2)	34.7 ± 4 (33.4–35.9)

Notes. Data are presented as mean ± standard deviation (95% confidence interval)

IV mean = intervention group mean, HC mean = historical group, DOL = day of life, PMA = postmentrual age, g = gramm; 95% CI = 95% confidence interval; ^1^*n* = 40, ^2^*n* = 35, ^3^*n* = 39, ^4^*n* = 41, ^5^*n* = 32, ^6^*n* = 37, ^7^*n* = 36; ^8^*n* = 41, ^9^*n* = 38, ^10^*n* = 29, ^11^*n* = 31

### Intervention

After reaching a weight above 1500 g, the infant was moved to the warming crib on the same day. The observation period for the primary outcome covered the period 48 h before to 72 h after transfer. Weaning from the incubator heat (Isolette 8000, Draeger, Germany) and humidity was not performed in the 48 h before the transfer. Infants were transferred to a warming crib (Babytherm 8000, Draeger, Germany; Lifetherm, Loewenstein medical, Germany) set at 37.0 °C on a gel mattress with a canopy. All infants were dressed in a bodysuit, a sweater, a romper, a wool cap, socks and were covered with two towels. The standard room temperature was 24 °C with a humidity of 45%. Skin temperature was continuously measured via a sternal and axillary probe (target temperature > 36.0 °C), rectal temperature was measured six times daily according to the clinical routine in our NICU (target temperature: > 36.5 °C). When the skin temperature was below the target, a rectal measurement was performed. When a rectal temperature < 36.5 °C was confirmed, the mattress temperature in the warming crib was increased by 0.5 °C up to a maximum of 38.0 °C. When the skin temperature increased to > 36.5 °C after this intervention, the warming crib was kept at the elevated temperature for 24 h. In case the temperature was still < 36.5 °C one hour after elevation or if the maximum temperature was reached, the infant was transferred back to the incubator. Kangaroo care was not limited by study participation. The time span of kangaroo care was excluded from continuous temperature analyses.

At the time of transfer, all infants were on full enteral feed with an intake of 160–170 ml/kg/d. Enteral feedings are introduced on the first day of life with a volume of 20–30 ml/kg/d. Feeds are advanced by 30 ml/kg/d if well tolerated. Up to a current weight of 1800 g infants receive either infant formula for very preterm infants or human milk with fortification.

### Monitoring

Vital signs were recorded using TrendFaceSolo 1.2-0 software (ixellence GmbH, Wildau, Germany) on a laptop from 48 h before transfer to 72 h after transfer. The data were exported as Microsoft Office Excel spreadsheets (Microsoft Corporation, Redmond, USA). Body weight was measured daily before feeding from 48 h before transfer to 72 h after transfer. The volume of feeding received via nasogastric tube or orally was documented daily.

### Data Collection for the historical cohort

In the historical comparison group (HC group), data of 42 VLBW preterm infants were retrospectively evaluated. These infants were born between December 2010 and October 2011 at the University Hospital Essen and were transferred to the warming crib when their weight reached 1700 g or more (refer to Table 1).

In the HC group, weight measurements were taken at birth, at the time of transfer from the incubator to the warming crib, at a PMA of 36.0 (± two days), at term equivalent age (± two days), and at the time of transfer to an unheated crib. Postmenstrual age was documented at birth, at the time of transfer to the warming crib, at the time of transfer to the unheated crib, and at discharge.

### Ethical approval

Ethical approval was obtained from the ethics committee of the Medical Faculty of the University of Duisburg-Essen (Approval Number: 12-5087-BO).

## Statistics

### Sample size calculation

Given the lack of reliable evidence in the literature regarding the target outcomes between early and late transfer and minimal concerns about the level of danger associated with the intervention, this study was conducted as a pragmatic pilot trial. Consequently, all eligible infants born between December 2012 and September 2014, whose parents had provided informed consent, were assigned to the intervention group. Based on previous admissions data, we estimated that 60 eligible infants would be born during the study period, and our goal was to include 42 of them (70%).

### Statistical analyses

We utilized the t-test for normally distributed data and the chi-square test for non-continuous data to compare variables. The significance level was set at *p* = 0.05. Since this study involved both prospective and historical group data, 95% confidence intervals (95% CI) were provided to facilitate a more comprehensive interpretation.

### Software

Statistical analysis was conducted using Microsoft® Excel® for MAC 2011 and IBM® SPSS Statistics Version 24.

## Results

**Fig. 1:**
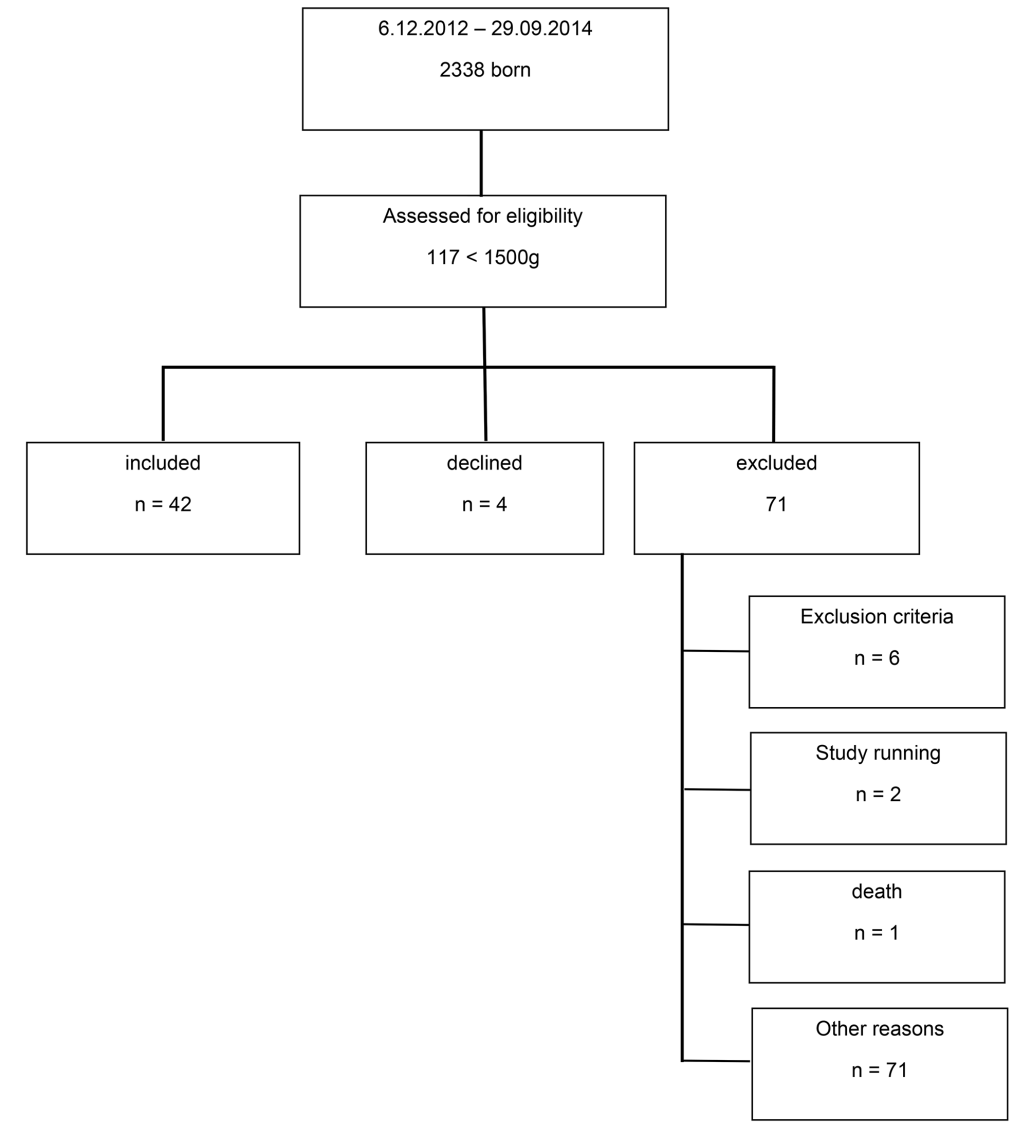
Probands. Exclusion criteria: invasive mechanical ventilation or noninvasive respiratory support like binasal continuous positive airway pressure (CPAP) with an additional oxygen requirement of > 0.25, failure to regain birth weight at the time of screening, ongoing phototherapy or indwelling central venous catheter /umbilical catheter. Study running: another patient was connected to the study monitor. Other reasons: Transfer, without information to study team, study team unavailable

During the study period, 117 infants weighing below 1500 g were born at our center, with 42 of them being recruited for the intervention (Fig. 1). PMA at birth and birthweight did not differ between the IV and the HC group (Table 1). The mean weight at transfer to the warming crib was 1558 ± 46 g in the IV group and 1710 ± 126 g in the HC group.

### Primary outcome in the IV Group

In the IV group, one infant exhibited hypothermia (rectal temperature of 36.3 °C), which was detected through routine measurement as part of standard care. Consequently, the temperature of the warming crib was raised to 38.0 °C without the need for transferring the infant back to the incubator.

### Secondary outcomes in the IV Group

The volume of oral feeding significantly increased on the day after transfer (24 ± 40 ml/kg/d before vs. 40 ± 48 ml/kg/d after transfer; *p* = 0.003) while the volume via gavage feeding significantly decreased from 126 ± 41 ml/kg/d to 112 ± 44 ml/kg/d (*p* = 0.008). The total volume of enteral feeding thus remained stable (*p* = 0.765). No significant differences were observed for daily weight gain before and after the transfer (Table 2).

### Secondary outcomes in comparison between the IV and HC groups

The mean body weight at the time of transfer to an unheated crib was significantly lower in the IV group (2057 ± 211 g vs. 2233 ± 251 g, *p* = 0.001). The mean PMA at this time point was also significantly lower in the IV group compared to the HC group (35.3 ± 1 weeks vs. 36.2 ± 2 weeks, *p* = 0.024) (Table 2). No significant differences were observed between the two groups for body weight at a PMA of 36.0 weeks, at a PMA of 40.0 weeks and at discharge, discontinuation of caffeine medication, discontinuation of gavage feedings, PMA at discharge, and length of hospital stay (Table 2).

### Protocol violations

In the IV group, 20 patients had a protocol violation because body weight was not measured daily during the test phase. A measurement taken every two days reflected the standard of care outside the study. In HC group, the children were weighed only every two days accordingly. Thus, in eight cases, weight was not available exactly on the day of transfer. Although the mean weight at the time of transfer in the HC group was significantly higher than in the IV group (1558 ± 46 g IV group vs.1710 ± 126 g HC group), 15 infants had a weight within the range of the intervention between 1500 g and 1650 g.

## Discussion

### Optimal timepoint for transfer

The optimal timepoint for transitioning a preterm infant from the incubator is a topic of ongoing debate. Clinical practice regarding this transfer largely depends on the individual judgment of attending nurses and physicians. Previous studies have typically studied weight limits between 1500 g and 1800 g [10, 4–9], as it is assumed that at this weight, there is sufficient subcutaneous adipose tissue to maintain normal body temperature. In our study, we observed one infant with hypothermia, but a transfer back to the incubator was not required. Additionally, we noted an increase in oral feeding volume on the day following the transfer, and both groups showed similar weight development at 36.0 week PMA, 40.0 weeks PMA, and at discharge.

### Methodological Variations

The comparability of studies on this subject is limited due to variations in methodologies. These differences include the type of crib used (with or without warming mattress or radiant heat), clothing of infants, and environmental conditions used (ambient temperatures ranged between 22 and 26 °C in previous studies) [3, 5–7, 9, 10]. In our study, the room temperature was maintained in the middle range at 24 °C. Weaning from respiratory support also exhibited considerable variation in previous studies, ranging from any respiratory support to CPAP and often occurring significantly later than at a current weight of 1500 g [5, 7, 9, 10]. To account for this, we included 12 infants with non-invasive respiratory support and a FiO2 < 0.25 in our study. Furthermore, the method of temperature measurement differed between studies, with the majority using intermittent axillary measurements [9, 10].The timing of measuring also varied between studies, ranging from hourly [10] or every three hours [9], and also varying over time. Notably, continuous skin temperature monitoring was not employed in any of the studies.

### Hypothermia and additional heat requirement

In our study, none of the infants in the study group required transfer back to the incubator due to hypothermia. Infants were transferred to the warming crib with standardized clothing and blankets, a practice commonly used in the majority of NICUs in Germany. In contrast, previous studies transferred infants to an unheated crib with the option to apply radiant heat in case of hypothermia [5, 7, 9]. Although some studies have demonstrated the safety of this approach [6–9], the study by New et al. reported higher rates of hypothermia compared to our findings [5].

The PMA at which additional heat by mattress was no longer necessary was significantly lower in the IV group than in HC group, aligning with the study by Barone et al. The temperature of the warming crib is gradually reduced, adapted to the child’s body temperature, until it is switched off at 32 °C and the gel mattress is changed to a foam matress. However, it is worth noting that smaller infants, some with complications, were more frequently transferred at a higher weight, a situation defined as delayed weaning in the study mentioned above [10].

### Length of Hospital Stay

While some previous studies have indicated a reduction of the length of hospital stay [6, 9, 10], our study, in line with the study by New et al., did not confirm this reduction [5]. The PMA at discharge did not differ significantly between the IV and HC groups. These results are consistent with the findings by West et al. [8], who compared transfer weights of 1600 g and 1500 g and achieved a comparable PMA at discharge to our IV group (38.3 (West) vs. 38.4 (IV) weeks). It is essential to recognize that factors other than temperature stability also influence the length of hospital stay, including feeding behavior, resolution of apnea of prematurity, and social factors. As an indication of this, our study demonstrates that the timing of transfer to the warming crib did not impact the time to achieve full oral feeds.

### Weight Development

Dollberg et al. highlighted an increased energy demand due to the transfer from the incubator [11], emphasizing the significance of monitoring weight development before and after transfer as an important safety aspect. In contrast, Berger et al. conducted a study comparing metabolic rates at transfer weights of 1500 g vs. 1600 g via indirect calorimetry and foundno significant differences in energy expenditure (2.9 ± 6.8 kcal/kg/d versus 1.1 ± 4.8 kcal/kg/d, *p* = 0.7) [3]. However, it is essential to note that other studies did not specifically examine the influence of transfer on weight gain.

In our study, we observed no significant difference in weight gain during the 48 h after transfer when comparing the IV and HC groups. Although there was a higher proportion of infants in the IV group (4/42 = 19% of infants) who did not gain any weight in the 48 h after transfer compared to the HC group (1/42 = 3.8% of infants), the overall difference in weight gain before and after transfer in the IV group was not statistically significant. In contrast, Barone et al. reported that infants transferred from incubator to warming crib at 1700 g compared to 1600 g gained significantly more weight (17.5 ± 4.8 g/kg/d vs. 11.1 ± 7.3 g/kg/d, p = < 0.001) [10]. It is worth noting that both groups in Barone’s study achieved lower weight gain, both before and after transfer, compared to our study (24 ± 17 g/kg/d before, 20 ± 16 g/kg/d after).

### Weight at different time points

Weight measurements at key time points, such as a PMA of 36.0 weeks (± 2 days), at discharge (± 2 days), and at term equivalent age (PMA 40.0 weeks ± 2 days), did not reveal any significant differences between the IV and HC groups.

In the study by West et al. [8], infants with transfer weights of 1600 g and comparable PMA at discharge had a lower weight at that time point compared to our IV group (West: 2320 ± 336 g vs. IV: 2768 ± 604 g). This discrepancy might be attributed to the additional heat provided by the warming crib in our study, as compared to an unheated crib as practiced in the study by West et al., resulting in a higher weight gain due to lower energy consumption. However, it is important to consider that better weight gain could have been achieved even before transferring due to different nutritional management, as suggested by the pre-transfer weight gain differences between the 1600 g group in West et al.’s study (15.9 g/kg/d) and the IV group in our study (24 ± 17 g/kg/d). Notably, our study’s weight gain exceeded the average weight gain of 16 g/kg/d in preterm infants with a current weight of 1000–1499 g [12].

### Enteral Feeding

The discontinuation of gavage feeding plays a pivotal role in determining the timing of discharge for premature infants. Our study observed a significant increase in oral feeding volume the day after transfer (*p* = 0.003), although the total volume remained consistent. This improvement in feeding may be attributed to facilitated nurse and parental handling in a warming crib. However, this positive effect did not lead to a faster weaning from gavage feeding in the IV group compared to the HC group. Schneiderman et al. demonstrated a negative correlation between increased weight at the time of transfer to the warming crib and the duration of time on gavage feeds. Specifically, they found that each 100 g increase in body weight resulted in a prolongation of 0.8 days on gavage feeds [6]. This suggests that the infant’s maturity plays a more significant role than weight alone. Furthermore, as indicated by Griffith et al., there is a significant negative correlation between the duration of gavage feeding and oral feeding success [13].

### Limitations

Several limitations are worth noting. Our study was conducted as a monocentric study due to structural differences, which may affect the generalizability of results. Randomization was not feasible due to the low birth rate of infants with a birth weight below 1500 g. Because only one study monitor and laptop were available, we could only include one infant at a time for the duration of the intervention phase (24 h before to 72 h after transfer). Additionally, protocol violations occurred in 20 patients of the IV group, where daily body weight measurements were not performed during the intervention phase. In the HC group, infants were weighed every two days according to the ward routine at that time, resulting in missing weight data in 8 cases on the day of transfer to the warming crib. Furthermore, one infant in the IV group did not have a recorded weight at the time of transfer. Although the mean weight at transfer in the HC group was significantly higher than in the IV group, 15 children in the HC group had weights between 1500 and 1650 g at time of transfer, which was within the limits that were intended for the intervention group. It is important to acknowledge the potential for bias in patient selection, as parents had to be proficient in the German language for informed consent, which lead to the exclusion of infants from non-German-speaking parents. Moreover, hygenic guidelines necessitated isolation or barrier precautions for infants with certain bacterial or viral colonization, rendering them ineligible for the study.

## Conclusion

In this prospective intervention study involving VLBW infants, we investigated the safety and efficacy of transferring neonates from incubators to warming cribs within a standardized weight range of 1500 g to 1650 g. Our findings indicate that this change in management, characterized by lower weight thresholds for transfer compared to a historical control group, is a safe approach for these vulnerable infants, with no infants requiring transfer back to the incubator due to hypothermia. Weight gain in the 48 h following transfer did not significantly differ between the intervention (IV) group and the historical comparison (HC) group, suggesting that this approach does not adversely impact short-term weight development. Additionally, while there was a significant increase in the volume of oral feeding the day after transfer, this did not accelerate the transition from gavage feeding in the IV group compared to the HC group. This underscores the importance of considering factors beyond weight, such as maturity, when determining the timing of transitioning to oral feeds. Our findings are consistent with previous studies examining weight gain, transfer weights, and safety; however, differences in methodologies, environmental conditions, and weaning protocols among studies limit direct comparisons. Notably, despite infants in the IV group being transferred to unheated cribs at an earlier PMA and lower weight compared to the HC group, we did not observe a significant reduction in the length of hospital stay. This suggests that factors beyond temperature stability, including feeding behavior and social factors, influence hospitalization duration. While acknowledging the limitations of our monocentric non-randomized study, we conclude that transferring VLBW infants to warming cribs within the specified weight range is a safe practice that does not adversely affect weight gain in the short term. Further research, including larger-scale studies, is warranted to provide comprehensive guidance on this aspect of neonatal care.

## Data Availability

The raw data supporting the conclusions of this article will be made available by the corresponding author, without undue reservation.

## Abbreviations

DOL: Day of life
GA: Gestational age
HC: Group historical group
IV: Group intervention group
PMA: Postmenstrual age
VLBW: Very low birth weight
